# Liposomal delivery enhances absorption of vitamin C into plasma and leukocytes: a double-blind, placebo-controlled, randomized trial

**DOI:** 10.1007/s00394-024-03487-8

**Published:** 2024-09-06

**Authors:** Martin Purpura, Ralf Jäger, Ashok Godavarthi, Dhananjaya Bhaskarachar, Grant M. Tinsley

**Affiliations:** 1 Increnovo LLC, 730 E Carlisle Ave, Whitefish Bay, WI 53217 USA; 2 Radiant Research Services Pvt. Ltd, Bangalore, 560058 India; 3 Department of Medicine, Shetty’s Hospital, 2nd F Main Rd, Kaveri Nagar, Bommanahalli, Bengaluru, Karnataka 560 068 India; 4 Energy Balance & Body Composition Laboratory, Department of Kinesiology & Sport Management, Texas Tech University, Lubbock, TX USA

**Keywords:** Liposome, Vitamin C, Bioavailability, Immune health

## Abstract

**Purpose:**

L-Ascorbic acid (vitamin C) is an essential water-soluble vitamin that plays an important role in various physiological functions, including immune health. The stability of vitamin C in the gastrointestinal tract its bioavailability is limited. This study aimed to investigate if a liposomal form of vitamin C can increase absorption compared to standard vitamin C.

**Methods:**

In a randomized, double-blind, placebo-controlled, crossover fashion, 19 males and 8 females (*n* = 27; 36.0 ± 5.1 years, 165.0 ± 6.9 cm, 70.6 ± 7.1 kg) ingested a single-dose of placebo (PLA), 500 mg vitamin C (VIT C), and 500 mg liposomal vitamin C (LV-VIT C, LipoVantage^®^, Specnova, LLC, Tyson Corner, VA, USA). Venous blood samples were collected 0, 0.5-, 1-, 1.5-, 2-, 3-, 4-, 6-, 8-, 12-, and 24-hours after ingestion and were analyzed for plasma and leukocyte vitamin C concentration.

**Results:**

VIT C and LV-VIT C demonstrated significantly greater Cmax and AUC_0 − 24_ in plasma and in leukocytes compared to placebo (*p* < 0.001). Additionally, LV-VIT C had significantly higher Cmax (plasma + 27%, leukocytes + 20%, *p* < 0.001) and AUC_0 − 24_ (plasma + 21%, leukocytes + 8%, *p* < 0.001) values as compared to VIT C.

**Conclusion:**

Liposomal formulation of vitamin C increases absorption into plasma and leukocytes.

**Trial Registration:**

Clinical Trials Registry - India (CTRI/2023/04/051789).

## Introduction

L-Ascorbic acid (vitamin C) is a water-soluble vitamin and essential for human health due to its pleiotropic functions related to its ability to donate electrons [1]. As a co-factor it facilitates collagen biosynthesis, carnitine, and catecholamine metabolism, and enables dietary iron absorption [2]. Vitamin C is also essential for wound healing and for the repair and maintenance of cartilage, bones, and teeth [3]. In addition, vitamin C plays a major role in immunity while protecting immune cells against oxidative stress generated during infections [4, 5].

For proper function as an effective antioxidant, vitamin C must be retained in the body at relatively high levels. Compared to many mammals which can easily produce vitamin C, humans do not have the capacity due to lacking the enzyme gulonolactone oxidase, which is required for vitamin C biosynthesis [6]. Therefore, humans must acquire vitamin C through diet. Dietary sources of vitamin C are fresh fruits and vegetables, like citrus fruits, berries, tomatoes, potatoes, bell peppers, and cruciferous leafy vegetables [7]. However, the actual dietary intake is lower than estimated due to the instability of vitamin C during cooking, storage, and food processing [8, 9]. The average adult stores 1.2–2.0 g of vitamin C within the body and at a daily consumption of approximately 140 mg the total body pool of vitamin C will be saturated [10]. The average half-life of vitamin C of 10–20 days results in a turnover of 1 mg/kg body weight. Consequently, vitamin C must be regularly consumed through diet or dietary supplementation to maintain the vitamin C pool in the body.

The pharmacokinetics of vitamin C in humans is highly complex in contrast to other low molecular weight ingredients [11]. The sodium-dependent vitamin C transporters (SVCTs) are responsible for the majority of intestinal uptake, tissue distribution, and renal reuptake. Plasma concentrations peak within 120 to 180 min after ingestion [12–14]. Vitamin C is absorbed and retained in cells and tissues, such as leukocytes. Therefore, due to its correlation with dietary intake of vitamin C, both plasma and leukocyte vitamin C concentrations have been identified as efficacy markers for its bioavailability [15].

The greatest challenge in the use and application of dietary supplements or food products with vitamin C is to maintain stability. Degradation processes of vitamin C easily take place in aqueous medium, at high pH, in the presence of oxygen and metal ions. Moreover, for sufficient oral supplementation of vitamin C, it must be protected against potential reduction due to degradation in the gut. Liposomes with one or more lipid bilayers using a hydrophilic–hydrophobic interface, can be used to reduce the degradation of vitamin C [16]. Liposomes are spherical vesicles of a bilayer of amphiphilic molecules like phospholipids. Since their first discovery in the 1960s by Alec Bangham, this process usually creates multilamellar vesicles (MLVs), which are concentric lipid bilayers separated by aqueous compartments [17, 18]. The first formulations were composed solely of natural lipids; at present they can include natural and/or synthetic lipids and surfactants. Since the 1960s, numerous methods have been developed to produce unilamellar liposomes of different sizes [19, 20]. The size of these nearly spherical lipid vesicles can range from a few nanometers to several micrometers. However, liposomes applied to medical use range between 50 and 450 nm [21]. Previous studies indicated potentially improved absorption of liposomal vitamin C compared to standard vitamin C; however, those studies were either not randomized nor double-blind, lacked a placebo group, used unpractical, extremely high amounts of vitamin C (4–36 g), or were of short duration (6–8 h) [16, 22–24].

The present study assessed the bioavailability of a single dose of 500 mg vitamin C in plasma and leukocytes following oral administration of vitamin C, liposomal vitamin C or placebo in male and female subjects over a period of 24 h. We hypothesized that vitamin C in liposomal form has better bioavailability in plasma and leukocytes compared to vitamin C and placebo.

## Materials and methods

### Participants

The study was approved by the Shetty’s Hospital Ethics Committee (registration number: ECR/918/Inst/KA/2017/RR-20) on April 6, 2023 (CL/VT/01/BA/2023) and the study was registered with the Clinical Trials Registry - India (CTRI/2023/04/051789). The study was conducted under the ICH guidelines for Good Clinical Practice at the Shetty’s Hospital, Department of Medicine, Bommanahalli, Bengaluru 560 068, Karnataka, India, following the ethical principles of the Declaration of Helsinki. The study was initiated on April 24, 2023, and study was completed on May 12, 2023.

Twenty-seven subjects were screened for this study. The sample size was determined based on previous studies of liposomal vitamin C studies [16]. Subjects enrolled in the study needed to meet the following inclusion parameters: 18–45 years of age, weighing at least 50 kg; have not been consuming any vitamin C-containing supplements or foods for 24 h prior to testing. Only healthy subjects were enrolled with no evidence of underlying disease during the pre-study screening, determined by medical history and physical examination through a physician, performed within 7 days prior to the commencement of the study. Exclusion criteria included: participants who are allergic to vitamin C; participants with resting hypertension (> 140/90 mmHg) and pulse rate below 50/min or more than 100/min; participants with or a prior history or presence of significant cardiovascular, pulmonary, hepatic, renal, hematological, gastrointestinal, endocrine, immunologic, dermatologic, neurological, musculoskeletal or psychiatric disease; participants who has been hospitalized or underwent surgery within the last 4 weeks; participants with a history of myocardial infarction, stroke, peripheral arterial disease, gastrointestinal bleeding, hepaticimpairment, asthma, renal impairment, epilepsy and intracranial hemorrhage; participants who have taken over the counter or prescribed medications including any enzyme modifying drugs within the last 14 days prior to the study; participants who have a history of alcoholism, drug abuse or smoking; and participants who have difficulty with donating blood or a history of difficulty in swallowing.

### Study materials

Vitamin C (VIT C) capsules, liposomal vitamin C capsules (LV-VIT C, LipoVantage^®^, Specnova, LLC, Tyson Corner, VA, USA) and placebo (PLA, maltodextrin) were acquired from Molecules Food Solutions Pvt Ltd, Kerala, India. Subjects ingested optically identical 1 hard gel capsules of each of the study materials per setting each yielding 500 mg of vitamin C or placebo. The vitamin C content was confirmed by an independent third-party analysis (Interfield Laboratories, Kochi, India; VIT C: 507 mg vitamin C per capsule, Certificate of Analysis # KH 96018/2023, July 26, 2023; LV-VIT C: 505 mg vitamin C per capsule, Certificate of Analysis # KH 96017/2023, July 26, 2023). The liposomal form of vitamin C consisted of ascorbic acid, sunflower lecithin with a proprietary ratio of phospholipids, gum arabic and alginate as sources of polysaccharides that make up the polar core of the liposome and are also located on the outside of the liposome. The liposomal structure was confirmed using transmission electron cryomicroscopy (CryoTEM).

### Study procedure

Prior to testing, each subject underwent screening and the consent visit to ensure eligibility and voluntary willingness to participate. After the written informed consent was obtained from the participants, their demographic data as well as medical history were recorded. A detailed physical examination including assessment of vital signs / parameters was done. All participants also underwent ECG and chest x-ray (PA view).

All participants provided blood for testing for hematological parameters such as hemoglobin, total leukocyte count, RBC count, platelet count, differential counts of neutrophils, lymphocytes, eosinophils, monocytes, basophils and ESR. All participants also underwent screening of biochemical parameters including renal function tests (blood urea nitrogen, serum uric acid, and serum creatinine), liver function tests (serum bilirubin, AST, (SGOT), ALT (SGPT), serum alkaline phosphatase, and serum albumin) and lipid profile (total cholesterol, HDL, LDL and VLDL). All participants also underwent testing of fasting blood glucose levels and routine urinalysis (color; appearance; specific gravity; pH; and presence of glucose, protein, bile salts, bile pigments, pus cells, epithelial cells, RBCs, casts, crystals, and bacteria). Serological screening included screening for HIV (HIV-1 and HIV2) and hepatitis B (HBsAg). Participants who were healthy without any signs or symptoms of any illness were enrolled in the study. Following enrollment, each volunteer completed 3 trials with 11 blood draws each in a randomized, double-blinded order separated by 3 days. Participants were not allowed any OTC medicines, herbal combinations, or prescription medications for at least 7 days prior to the study drug administration until the study completion period. Participants were also restricted from consuming alcohol, citrus fruits, smoking cigarettes, chewing tobacco, or consuming any caffeine containing product within 36 h of in-housing until all blood draws were completed. The diet was restricted such that no vitamin C containing products were consumed for at least 24 h prior to administration of the investigational products until all blood draws were completed. Participants were also restricted from donating blood from the day of the study enrolment until the end of the study; and from carrying out any strenuous physical exercise during the study period. During each trial, each volunteer reported to the laboratory in the morning following a 12‒hour overnight fast (except for water). The participants were admitted in-house into the study center after they underwent successful screening. The study materials were administered under supervision and blood was drawn at pre-determined intervals during the in-house admission. The participants were allowed to go out of the in-house facility during the washout periods and the participants again were admitted in-house for second testing where they again received the study materials according to the sequence of allocation. The participants then underwent a second washout period where they again were allowed to go outside of the in-house facility. The participants again were in-housed for the third time where they received the study materials as per the schedule and finally the participants finished the study after blood was withdrawn as per the study protocol.

**Fig. 1:**
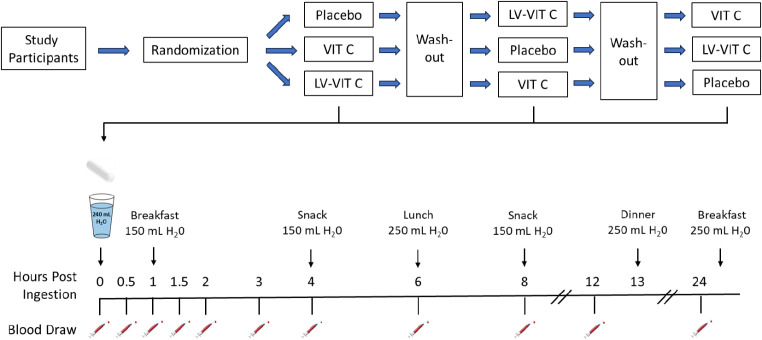
Schematic overview of research design

This study was conducted as a randomized, double-blind, placebo-controlled crossover study. All eligible participants were equally randomized into three groups of 9 participants each by simple randomization into group 1, group 2 and group 3. Participants in group 1 were sequentially allotted to treatment A, B and C, participants in group 2 sequentially were allotted to treatment B, C and A; and participants in group3 sequentially were allotted to treatment C, A and B (see Fig. 1). To maintain blindness of the study treatments, all the test products were identical in appearance. Study materials were coded centrally with randomization numbers as per the computer-generated randomization schedule.

Subjects were not allowed to drink water between 1 h before to 1 h after the ingestion of the Investigational products, except while dosing. The test materials were administered orally in sitting posture, with 240 mL of water at ambient temperature, as a single dose, as per the randomization code list. Participants were checked-in to the clinical facility the evening prior to the administration of the test materials (Day 0) and were given standardised meal for dinner consisting of chapathi made of wheat flour, 1 bowl of rice (200 g), fried vegetable (50 g), sambar (stew of lentil) and water (500 mL). On Day 1, food was provided at 1-hour (breakfast: idly, made of rice flour and black lentil (200 g), chutney, made of grated coconut and peanuts, and water (150 mL)); 4-hours (snack: coffee or tea (one cup: 75 mL), biscuit (50 g) and water (150mL)); 6-hours (lunch: 1 bowl of rice (300 g), fried vegetable (50 g), Sambar (stew of lentil), and water (250 mL)); 8-hours (snack: coffee or tea (one cup: 75 mL), biscuit (50 g) and water (150 mL)); and 13-hours (dinner: chapathi, made of wheat flour, 1 bowl of rice (200 g), friend vegetable (50 g), Sambar (stew of lentil) and water (250 mL)) post administration. On Day 2, the final blood draw was performed by 24-hours after the dosing followed by standard breakfast (Idly made of rice flour and Black lentil (200 g), chutney made of grated coconut and peanuts and water (250 mL)) before the participant left the clinical facility.

During each visit, the subjects were seated comfortably while a catheter was introduced into a forearm vein by a qualified phlebotomist. After equilibration, a baseline blood sample was collected, and one of three treatment dosages (LV-VIT C, VIT C, or PLA) was administered with water. Blood samples were then drawn at 0.5-, 1-, 1.5-, 2-, 3-, 4-, 6-, 8-, 12-, and 24-hours intervals following test product administration. Each subsequent trial was separated by at least 3 days as a washout period and followed identical study procedures, except for the consumption of a different vitamin C or placebo formulation (see Fig. 1).

### Adverse event/safety monitoring

During the entire period, the participants were monitored for any adverse events. Vital signs: blood pressure (sitting or semi-supine position); radial pulse rate; temperature; respiratory rate was assessed on admission, and at different intervals: prior to dosing at 0 h, 15 min, 30 min, 1 h, 2 h, 4 h, 8 h, 12 h, and 24 h prior to the time of participant checkout. Vital signs were also recorded 2 h prior to the administration of the study materials. A window period of ± 15 min from the scheduled time point was allowed for post dose recording of vital signs. Physical examination was performed at admission and 1, 2, 4, 8, 12 and 24 h after intake of the study materials.

### Sample collection

During each timepoint, 10 mL of blood were drawn off the catheter into vacutainer tubes. Blood tubes were centrifuged at 5000×g for 15 min. Following centrifugation, plasma and buffy coat (leukocytes) were separated into labeled microcentrifuge tubes using micropipette as individual aliquots. The samples were stored in a ‒80 °C freezer until analysis and thawed only once prior to the respective analysis to avoid degradation.

### Sample preparation and analysis

Samples were prepared for HPLC/MS/MS analysis by using Solid Phase Extraction (SPE) method. At the time of analysis, the samples were removed from the deep freezer and kept in the room temperature and allowed it to thaw. SOLA CX SPE Columns C18-(50 μm, 70 A) solid phase extraction cartridge was conditioned with methanol and water sequentially. To this, 250 µL aliquot of plasma containing vitamin C was pipetted and the SPE cartridge was loaded. The cartridge was washed with 1.0 mL of methanol. The drug was eluted from the cartridge using water and 300 µL of mobile phase. The samples were injected into the HPLC system for analysis with UV absorbance at 243 nm. The following parameters were assessed: area under the curve (AUC_0 − 24_), maximum observed concentration (Cmax), time of maximum concentration (Tmax), mean and percentage changes of vitamin C from baseline (0 h) in plasma and leukocytes.

### Statistical analysis

Cmax and AUC_0 − 24_ data were analyzed using one-way analysis of variance with repeated measures, with condition specified as the within-subjects factor. Data were grouped by condition and inspected for extreme outliers (i.e., values above Q3 + 3 x IQR or below Q1–3 x IQR), normality, and sphericity. Outliers were detected only in the PLA condition (*n* = 1 for leukocyte Cmax, *n* = 2 for leukocyte AUC and *n* = 1 for plasma AUC). As these points were only present in PLA and were not influential in the overall outcomes, all data points were retained in the analysis. Normality was examined via quantile-quantile plots grouped by condition, and data were approximately normally distributed. When sphericity was violated based on Mauchly’s test, the Greenhouse-Geisser correction was applied. Following a statistically significant effect of condition, post hoc pairwise t-tests were performed, using the Bonferroni correction for multiple comparisons. The generalized eta-squared (η2G) effect size was calculated to accompany the one-way analysis of variance test, and Cohen’s d effect sizes were calculated to accompany each pairwise t-test. Due to the nature of the data, Tmax values were analyzed using the non-parametric Friedman test, accompanied by Kendall’s W effect size. Following a statistically significant effect of condition, post hoc Wilcoxon signed-rank tests were performed. For all tests, statistical significance was accepted at *p* < 0.05. Data are presented as mean ± SD unless otherwise noted. Data were analyzed in R software v. 4.3.1 [25] with the *rstatix* package v. 0.7.2 [26].

## Results

### Subjects

**Fig. 2:**
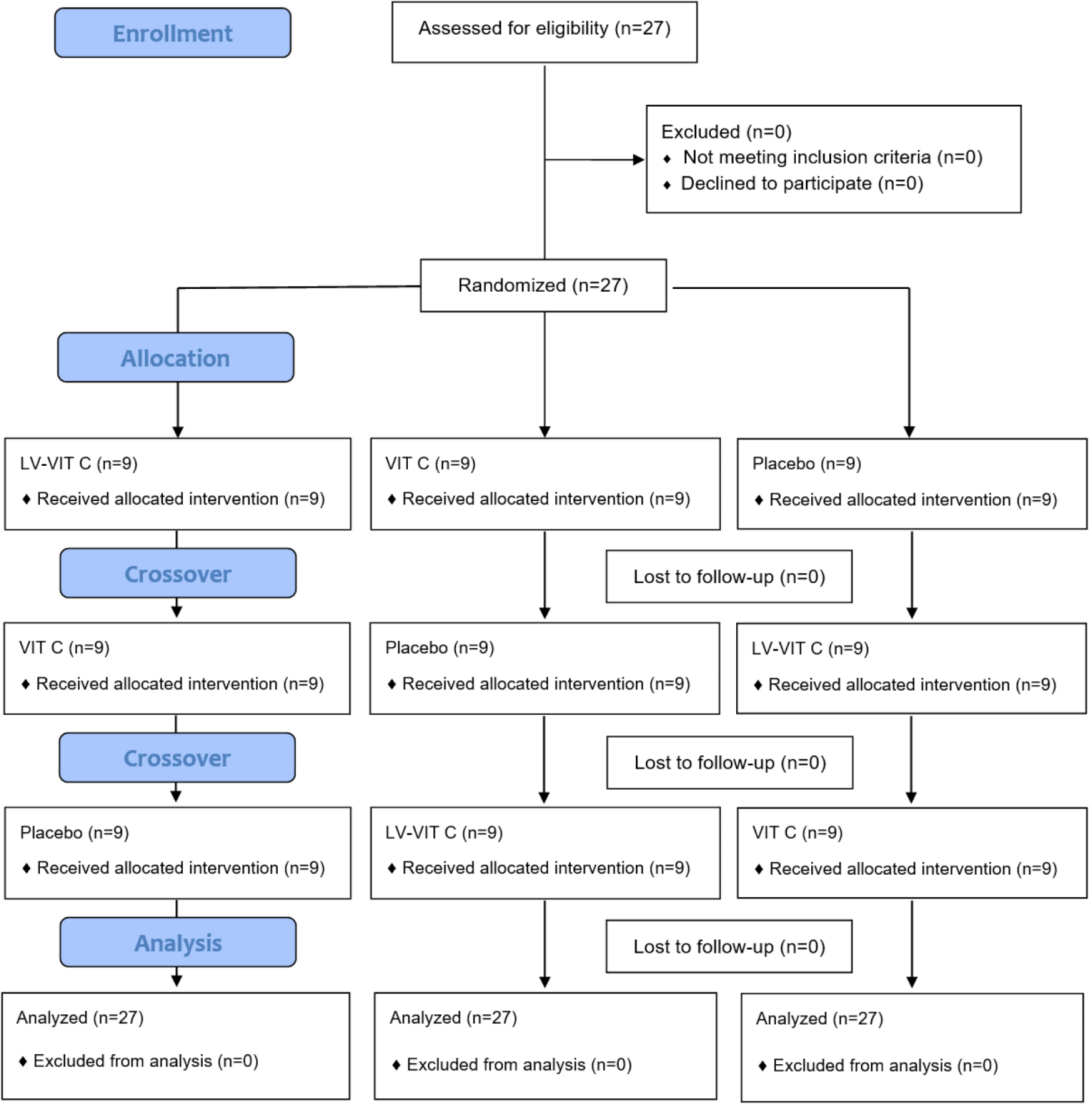
CONSORT flow diagram

Twenty-seven participants (8 F, 19 M) completed the present study ([mean ± SD] age 36.0 ± 5.1 y, height 165.0 ± 6.9 cm, weight 70.6 ± 7.1 kg, BMI 25.9 ± 1.6 kg/m^2^, systolic blood pressure 120.0 ± 7.1 mmHg, diastolic blood pressure 77.0 ± 7.8 mmHg) (Fig. 2).

### Adverse events

None of the participants reported any adverse events during or after the study period. All the participants tolerated the investigational products equally well without any evidence of any abnormality of vital signs or any abnormality during physical examination, which were assessed at different time intervals after administration of the study medications.

### Plasma

**Fig. 3:**
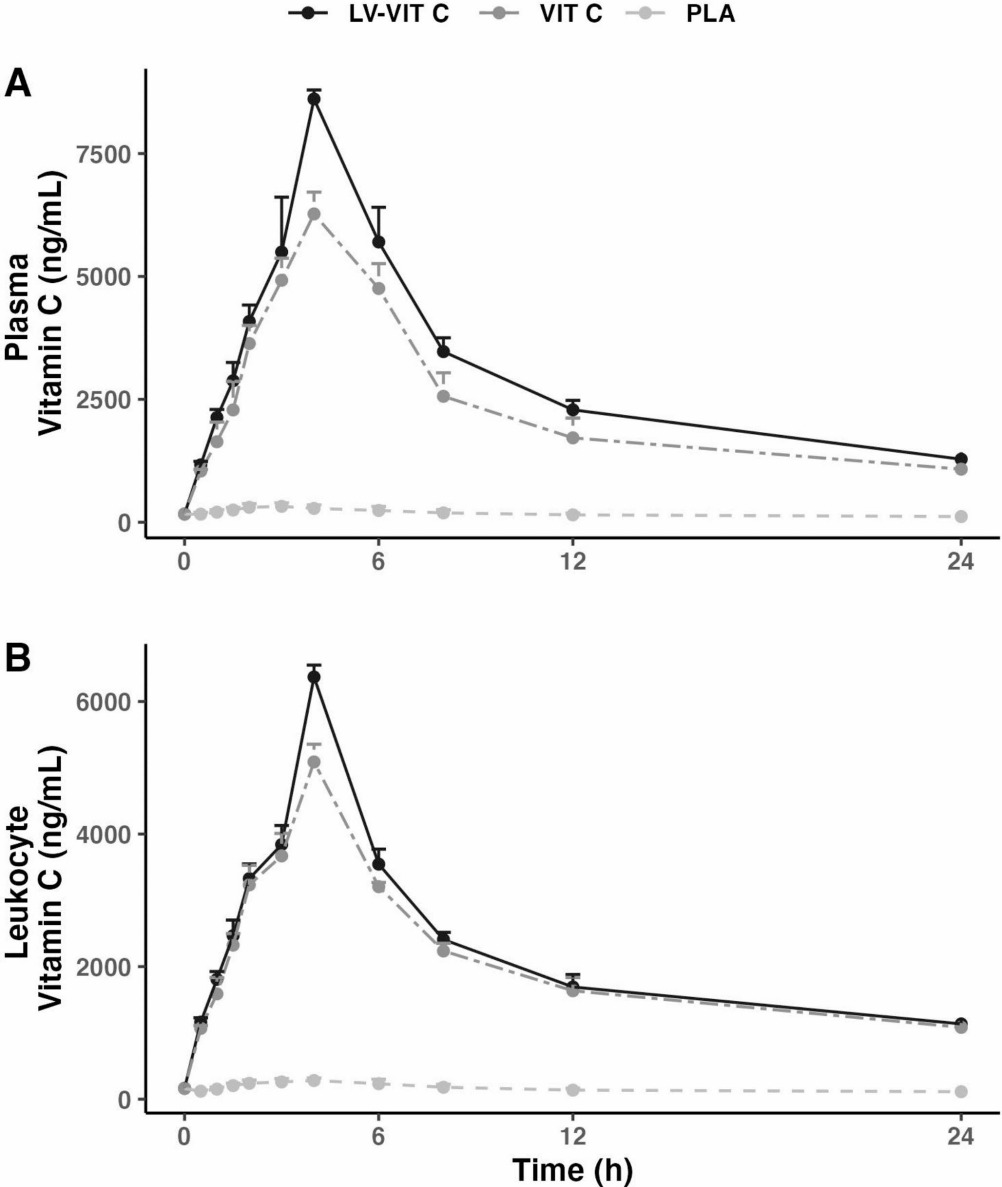
Effects of liposomal delivery on vitamin C absorption. Raw concentrations of vitamin C in plasma (**A**) and leukocytes (**B**) are displayed for 24 h following ingestion of liposomal vitamin C (LV-VIT C), standard vitamin C (VIT C), or placebo (PLA)

**Fig. 4:**
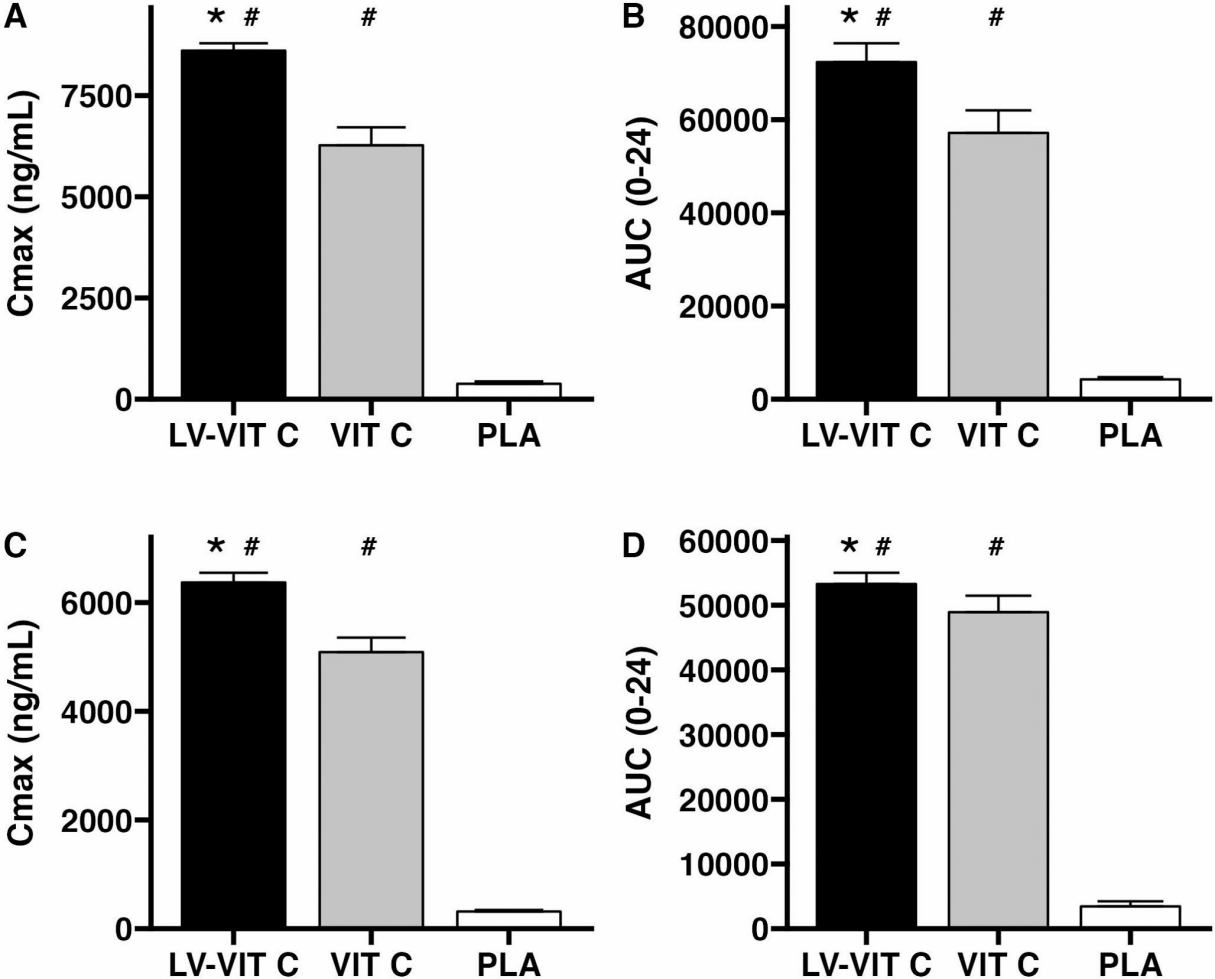
Pharmacokinetic comparison of vitamin C absorption. In plasma (**A**, **B**) and leukocytes (**C**, **D**), a significant benefit of liposomal delivery (LV-VIT C) on vitamin C maximal concentrations (Cmax) and area under the curve over 24 h (AUC_0 − 24_) was observed. #=significant difference compared to placebo (PLA). *=significant difference compared to standard vitamin C (VIT C)

Plasma Cmax significantly differed between conditions (LV-VIT C 8,610 ± 182 ng/mL, VIT C 6,271 ± 444, PLA 379 ± 59; *p* < 0.001, η2G = 0.99; Fig. 3A). Additionally, each condition significantly differed based on post hoc *t*-tests (*p* < 0.001 for each comparison; Fig. 4A). Cohen’s d effect sizes were 13.2 for VIT C v. PLA, 46.0 for LV-VIT C v. PLA, and 4.9 for LV-VIT C v. VIT C. Expressed as percentages, plasma Cmax was 2,174% higher with LV-VIT C than PLA and 1,556% higher with VIT C than PL, on average. The percent difference between LV-VIT C and VIT C was 27%, with higher Cmax values in the LV-VIT C condition.

For plasma Tmax, a significant effect of condition was observed (*p* < 0.001, Kendall’s W = 0.40) For LV-VIT C and VIT C, all participants demonstrated a Tmax value of 4 h. For PLA, Tmax values were 3 ± 1 h (median ± IQR). Post hoc tests indicated significant differences between LV-VIT C and PLA (*p* = 0.048) and between VIT C and PLA (*p* = 0.048).

Plasma AUC_0 − 24_ significantly differed between conditions (LV-VIT C 72,358 ± 4,044 ng/mL * 24 h, VIT C 57,152 ± 4,846, PLA 4,242 ± 486; *p* < 0.001, η2G = 0.98), with post hoc *t*-tests indicating a difference between each comparison (*p* < 0.001 for each; Fig. 4B). Cohen’s d effect sizes were 10.9 for VIT C v. PLA, 16.2 for LV-VIT C v. PLA, and 2.8 for LV-VIT C v. VIT C. Expressed as percentages, LV-VIT C and VIT C exhibited values that were 1,605% and 1,247% higher than PL, on average, with a 21% difference between LV-VIT C and VIT C conditions.

### Leukocytes

Leukocyte Cmax significantly differed between all conditions (LV-VIT C 6,369 ± 179 ng/mL, VIT C 5,088 ± 267, PLA 315 ± 29; *p* < 0.001, η2G = 0.99; Fig. 3B). Additionally, each condition significantly differed based on post hoc *t*-tests (*p* < 0.001 for each comparison; Fig. 4C). Cohen’s d effect sizes were 17.7 for VIT C v. PLA, 33.0 for LV-VIT C v. PLA, and 6.2 for LV-VIT C v. VIT C. Expressed as percentages, leukocyte Cmax was 1,922% higher with LV-VIT C than PLA and 1,515% higher than VIT C than PL, on average. The percent difference between LV-VIT C and VIT C was 20%, with higher Cmax values in the LV-VIT C condition.

For leukocyte Tmax, no significant effect of condition was observed (*p* = 0.72, Kendall’s W = 0.01) For LV-VIT C and VIT C, all participants demonstrated a Tmax value of 4 h. For PLA, Tmax values were 4 ± 0.5 h (median ± IQR).

Leukocyte AUC_0 − 24_ significantly differed between conditions (LV-VIT C 53,277 ± 1,738 ng/mL * 24 h, VIT C 48,922 ± 2,548, PLA 3,439 ± 789; *p* < 0.001, η2G = 0.99), with post hoc *t*-tests indicating a difference between each comparison (*p* < 0.001 for each; Fig. 4D). Cohen’s d effect sizes were 17.8 for VIT C v. PLA, 26.2 for LV-VIT C v. PLA, and 1.6 for LV-VIT C v. VIT C. Expressed as percentages, LV-VIT C and VIT C exhibited values that were 1,449% and 1,323% higher than PL, on average, with an 8% difference between LV-VIT C and VIT C conditions.

## Discussion

This randomized, double-blind, placebo-controlled, crossover study examined the bioavailability of liposomal and standard vitamin C following a single oral administration compared to placebo into plasma and leukocytes. Liposomal vitamin C significantly increased maximum plasma (+ 27%) and leukocytes (+ 20%) concentrations compared to non-liposomal vitamin C over a period of 24 h.

A first pilot study investigating phosphatidylcholine-based liposomal vitamin C used a single blind design, measuring plasma levels in just two subjects. Following oral single ingestion of a high dose (5 g, 20–36 g) of vitamin C, blood draws were taken over 6 h. At the 5 g dose, the liposomal and standard formulation showed similar plasma responses. Liposomes are absorbed from the gut into the liver, before being released into the blood resulting in a slower onset of peak levels in the 20 g dose [23].

Orally administered vitamin C is less bioavailable compared to infusing vitamin C. Encapsulating vitamin C in liposomes increases absorption of a single 4 g dose of vitamin C compared to standard vitamin C, however, bioavailability is still less than intravenous administration [24]. A high dose (10 g of vitamin C) of phosphatidylcholine-based liposomal vitamin C, with a concentration of 20% by weight, administered as a liposomal suspension showed increased absorption compared to an aqueous solution of vitamin C [16]. High-dose (5 g of vitamin C) phosphatidylcholine-based liposomal vitamin C, with a concentration of 66% by weight, showed a significant increase in plasma vitamin C levels in an open-label study [27]. Our study used a low dose of vitamin C (500 mg) compared to the previous studies investigating liposomal vitamin C formulations (4–36 g), an amount that is more in line with the generally recommended intake of vitamin C for healthy people, and for groups that are more likely to be at risk of vitamin C inadequacy. In addition, this is the first study with liposomal vitamin C following rigorous scientific standards for clinical studies, specifically a randomized, double-blind, placebo-controlled design.

Vitamin C is linked to immune health which depends on the incorporation of vitamin C into leukocytes. Our study showed an increase in leukocyte vitamin C concentration with a liposomal form of vitamin C in addition to increases in plasma. Previously, 1,000 mg of a non-liposomal commercial form of vitamin C that contained calcium ascorbate and vitamin C metabolites, including dehydroascorbate, calcium threonate, and 4-hydroxy-5-methyl-3(2 H)-furanone was shown to increase vitamin C levels in leukocytes from baseline values; however, similar increase in vitamin C levels was noted from standard vitamin C and placebo. In addition, the formulation failed to increase plasma concentrations compared to standard vitamin C [28].

## Conclusions

Liposomal vitamin C formulation (LipoVantage^®^) significantly increased plasma and leukocyte vitamin C levels compared to standard non-liposomal vitamin C and placebo. This may be due to increased absorption of encapsulated vitamin C from the gastrointestinal tract, protection against enzymatic degradation in the gastrointestinal tract and the plasma and enhanced penetration and retention by the leukocytes.

## Data Availability

The datasets generated during the current study are available upon request.
